# COVID-19 and stroke—A global World Stroke Organization perspective

**DOI:** 10.1177/1747493020923472

**Published:** 2020-04-29

**Authors:** Hugh S Markus, Michael Brainin

**Affiliations:** 1 Department of Clinical Neurosciences, University of Cambridge, Cambridge, UK; 2 Department for Clinical Neuroscience and Preventive Medicine, Danube University Krems, Krems, Austria

**Keywords:** COVID-19, stroke, pandemic, World Stroke Organization, Intensive Care Unit, healthcare systems

## Abstract

The COVID-19 pandemic affecting all parts of the world is having huge implications for stroke care. Not only do stroke patients appear to be more susceptible to severe infection, but the pandemic is having major implications on how we deliver stroke care, while ensuing safety of both our patients and health care professionals. COVID-19 infection itself has also been described as a risk factor for stroke. The World Stroke Organization has been monitoring the impact of the pandemic globally, and has identified an initial marked fall in stroke presentations as well as a widespread impact on stroke services. The pandemic is changing the way we deliver care, and has highlighted the enormous potential of telemedicine in stroke care.

The COVID-19 pandemic affecting all parts of the world is having major implications for stroke care, both direct and indirect. Stroke patients themselves appear particularly susceptible to developing complications and death when suffering COVID-19 infection, as highlighted in a pooled analysis of the available data published by Aggarwal et al. in International Journal of Stroke (IJS).^1^ They therefore need to be protected from contact with infected individuals. However, the pandemic is having a much wider impact on stroke care, with significant pressures on delivery of stroke services, meaning the outcome of patients presenting with stroke but without COVID-19 may be worse than at normal times.

## The impact on the pandemic on acute stroke services

The World Stroke Organization (WSO) has been monitoring experiences across the globe.^2^ While a small minority of countries are managing to maintain a full range of acute stroke services, most have seen significant service reorganization. WSO members report reallocation of neurology and stroke beds including Intensive Care Unit facilities to COVID-19 patients necessitating a move of stroke units to less optimal accommodation and redeployment of stroke physicians, nurses, and other stroke healthcare-related workers to look after COVID-19 patients.^2,3^ The ability to offer endovascular treatments has been reduced or stopped in many units. Even intravenous thrombolysis is under threat with at best service pressures and delays imposed by managing potentially infected patients resulting in increased door to needle times, and at the worst stroke patients missing the therapeutic window altogether due to delays in hospital admission or referrals, or patients preferring not to enter the hospital at all. In preparation for the suspected influx of COVID-19 patients, many healthcare systems have reduced or stopped provision of “non-urgent” care which will particularly impact on stroke prevention and follow-up. Even urgent interventions such as carotid endarterectomy have been put on hold due to priority being given, quite understandably, to utilizing bed and High Dependency Unit (HDU) and Intensive Therapy Unit (ITU) resources for managing COVID-19 patients.

## The apparent reduction in acute stroke cases

All countries are facing challenges, and while these differ according to the local healthcare system, a clear message coming out from the WSO survey across multiple countries including Chile, Colombia, Iran, Greece, UK, Belgium, and Italy is that there has been a sharp reduction in the number of acute stroke admissions. This is likely to be due to a reduction in admissions of patients with milder stroke, perhaps due to fears of infection if they are referred to hospital during times of social distancing and lockdown. However, in some countries, the reported number of acute stroke admissions has fallen by as much as 50% and even 80%,^2^ suggesting that many patients with moderate and even severe stroke, who could benefit from acute stroke therapies, are not being admitted. Furthermore, even patients with milder stroke may benefit from acute stroke therapies, and also from early assessment and implementation of secondary preventative measures, which have been shown to have a major impact on stroke burden; the stroke recurrence rate after Transient Ischaemic Attack (TIA) or minor stroke is about 10% in the first week.^4^ These alarming figures have led to alerts from both medical and patient organizations emphasizing the need for acute stroke services to be maintained even in these difficult times, and for patients and their family doctors to ensure that referral to hospital for acute stroke continues. The importance of preserving care for critical conditions such as stroke, and for strategies to ensure this continues, have been highlighted in formal advice from national bodies worldwide such as the English National Institute of Clinical Excellence (NICE).^5^

## Does COVID-19 increase stroke risk?

The true relationship between COVID-19 and stroke incidence remains to be determined. Despite the fall in stroke admissions, it has been suggested that COVID-19 infection itself may cause stroke. In a study of 214 COVID-19 cases from Wuhan, China, where the pandemic was first identified, 36.4% had neurological symptoms, and these were more frequent in patients with severe disease.^6^ The most common symptoms identified were dizziness (16.8%) and headache (13.1%), while anosmia occurred in 5.1%. Stroke occurred in six cases (2.8%), but all but one case were seen in the severe infection group; most were ischemic but one case of intracerebral hemorrhage occurred. A number of potential mechanisms by which COVID-19 might increase stroke risk have been identified, but not yet proven to increase risk. These include hypercoagulability as evidenced by raised D-dimer levels,^6^ exaggerated systemic inflammation or a “cytokine storm” which is a hallmark of severe disease,^7^ and cardio embolism from virus-related cardiac injury.^8^ Cardiac injury appears to be a prominent feature of the disease, occurring in 20–30% of hospitalized patients.^8^ Direct viral invasion of the nervous system could also contribute,^6^ and there may be direct cerebral consequences, such as the recent description of COVID-19-associated acute hemorrhagic necrotizing encephalopathy, similar to that previously described with Severe Acute Respiratory Syndrome Coronavirus 2.^9^ Conversely, there could also be associated factors which could potentially reduce stroke incidence. For example, a striking reduction in pollution has been reported in multiple countries during the pandemic secondary to lockdown.^10^ Pollution is associated with an increased cardiovascular and stroke risk, and a reduction could be potentially protective against stroke.^11^ Much research is required to look further into the relationship between COVID-19 and stroke prevalence and outcome.

## Sharing expertise and best practice guidelines

There has been widespread sharing of experiences worldwide, and we are very grateful for the rapid publication of the Chinese experience, which preceded that of the rest of the world by a number of weeks. Best practice guidelines provide guidance as to how to treat stroke in the context of the pandemic, while safeguarding healthcare workers. Such guidance is published in the IJS by an international collaboration from 18 countries, with members selected on the basis of previous experience not only in COVID-19 but also in previous coronavirus infection outbreaks such as Middle East Respiratory Syndrome and Severe Acute Respiratory Syndrome.^12^ This provides useful practical advice on how to identify COVID-19 patients and how to provide optimal stroke care while ensuring maximum protection for staff involved. How different healthcare systems adapt is likely to vary. For example, in better funded situations separate angiographic and assessment pathways are being devoted to COVID-19 and non-COVID-19 patients, but this is not possible in many countries.

Continuing to provide management for those patients with minor stroke and TIA who do not necessarily require admission to hospital also presents challenges. Careful assessment needs to be made of the risk:benefit ratio of asking an elderly frail patient with TIA to attend hospital. Many healthcare systems have suggested that all TIAs should initially be assessed by telemedicine, as illustrated by the English NICE guidelines.^5^ Even in those situations where carotid endarterectomy is not widely available during the pandemic, it is important that simple secondary prevention measures such as aspirin, or anticoagulation when required, treatment of risk factors, and adoption of lifestyle measures are implemented.

## The potential of telemedicine

Telemedicine offers many opportunities during the current crisis, and we may learn improved management strategies using such technologies, which can be continued into routine care once the pandemic is over.^13^ Stroke has led the way in telemedicine for acute assessment for thrombolysis. This continues to be a central part of stroke care particularly in rural settings, and an increased use of telemedicine may assist during the current pandemic. This solution avoids the use of needed Personal Protective Equipment, allows a reasonable stroke evaluation, avoids unnecessary inter-facility transfers, and reduces exposure risk for the stroke team.^14^ The National Institute of Health (NIH) Stroke Scale can be performed efficiently via telemedicine. Video telemedicine is superior to telephone, but telephone consultation is superior to no consultation.^14^

Telemedicine is also well suited not only for acute stroke but also for TIA and stroke out-patient follow-up. It allows follow-up while maintaining the social isolation put in place in many countries. This can be performed by telephone, but better patient interaction and limited examination can be performed by video teleconferencing. Video teleconferencing has been shown to be well suited to out-patient follow-up of patients with rare stroke diseases, with excellent patient experience similar to that for in-person appointments.^15^ Of relevance to a wider stroke population, many of whom are elderly or have cognitive or neurological disability, patient and clinician experiences were equally good for patients with cognitive impairment whether they were seen in-person or via video teleconferencing.^15^

## Impact on developing countries

The WSO represents stroke throughout the world and a major aim is to improve stroke care in developing countries. The potential impact of COVID-19 on developing countries is particularly concerning. Many have not only much less developed stroke services but also less developed acute medical services to manage the COVID-19 pandemic, including a major shortage of ventilated intensive care beds, which are vital to support patients throughout the acute respiratory distress syndrome period. It has also been suggested that certain ethnic groups may be more susceptible to COVID-19 infection with increased severity of infection and mortality; early reports have described increased disease severity in black and Asian people in the UK,^16^ and in African Americans in the USA.^17^ It remains uncertain as to whether this is due to confounders such as lower socioeconomic status, comorbidities, and different levels of exposure. Genetic factors themselves may predispose some individuals to particularly severe infection but as yet this remains unproven; it is a subject of major international collaborative research such as the COVID-19 Host Genetics Initiative.^18^

## The future

The next few months will be a time of great uncertainty both for stroke care and for global health. Healthcare systems have adapted very rapidly to implement systems for COVID-19 care, but it is important that we ensure that the highly effective stroke therapies we have today continue despite these service reorganizations. There has already been enormous sharing of expertise throughout the world to help each other implement best practice, which is a major achievement. Even when the COVID-19 pandemic is over, the consequences for stroke are likely to persist. Economic factors are well recognized as a risk for stroke incidence and severity, and the economic impact of the COVID-19 pandemic is predicted to be enormous.

## Dedication

This article is dedicated to the memory of Professor John Norris, a pioneer in developing stroke care particularly in Canada, advancing stroke research particularly in carotid artery disease and dissection, and a mentor to many, who was a victim of the COVID-19 pandemic.
